# Pandemic influenza preparedness in the WHO African region: are we ready yet?

**DOI:** 10.1186/s12879-018-3466-1

**Published:** 2018-11-14

**Authors:** Evanson Z. Sambala, Tiwonge Kanyenda, Chinwe Juliana Iwu, Chidozie Declan Iwu, Anelisa Jaca, Charles S. Wiysonge

**Affiliations:** 1 Cochrane South Africa, South African Medical Research Council, Box 19070, Cape Town, PO 7505 South Africa; 2 Vaccines for Africa Initiative, Division of Medical Microbiology and Institute of Infectious Disease and Molecular Medicine, University of Cape Town, Cape Town, South Africa; 3 Division of Health Systems and Public Health, Department of Global Health, Faculty of Medicine and Health Sciences, Stellenbosch University, Cape Town, South Africa; 4 Department of Biochemistry and Microbiology, University of Fort Hare, Alice, South Africa; 5 Division of Epidemiology and Biostatistics, School of Public Health and Family Medicine, University of Cape Town, Cape Town, South Africa; 6 Centre for Evidence-Based Health Care, Division of Epidemiology and Biostatistics, Department of Global Health, Faculty of Medicine and Health Sciences, Stellenbosch University, Cape Town, South Africa

**Keywords:** National preparedness plans, Pandemic influenza, Africa, Quality of the plans, Surveillance, Containment, Communication, Ethical framework, Treatment

## Abstract

**Background:**

Prior to the 2009 pandemic H1N1, and the unprecedented outbreak of Highly Pathogenic Avian Influenza (HPAI) caused by the H5N1 virus, the World Health Organization (WHO) called upon its Member States to develop preparedness plans in response to a new pandemic in humans. The WHO Member States responded to this call by developing national pandemic plans in accordance with the International Health Regulations (IHR) to strengthen the capabilities of Member States to respond to different pandemic scenarios. In this study, we aim to evaluate the quality of the preparedness plans in the WHO African region since their inception in 2005.

**Methods:**

A standard checklist with 61 binary indicators (“yes” or “no”) was used to assess the quality of the preparedness plans. The checklist was categorised across seven thematic areas of preparedness: preparation (16 indicators); coordination and partnership (5 indicators); risk communication (8 indicators); surveillance and monitoring (7 indicators); prevention and containment (10 indicators); case investigation and treatment (10 indicators) and ethical consideration (5 indicators). Four assessors independently scored the plans against the checklist.

**Results:**

Of the 47 countries in the WHO African region, a total of 35 national pandemic plans were evaluated. The composite score for the completeness of the pandemic plans across the 35 countries was 36%. Country-specific scores on each of the thematic indicators for pandemic plan completeness varied, ranging from 5% in Côte d’Ivoire to 79% in South Africa. On average, preparation and risk communication scored 48%, respectively, while coordination and partnership scored the highest with an aggregate score of 49%. Surveillance and monitoring scored 34%, while prevention and containment scored 35%. Case investigation and treatment scored 25%, and ethical consideration scored the lowest of 14% across 35 countries. Overall, our assessment shows that pandemic preparedness plans across the WHO African region are inadequate.

**Conclusions:**

Moving forward, these plans must address the gaps identified in this study and demonstrate clarity in their goals that are achievable through drills, simulations and tabletop exercises.

## Background

Pandemic influenza is a rare disease caused by a novel influenza virus, a subtype that has the capability to cause sustained human-to-human transmission and to which the population has no or little immunity [1]. Historically, there have been 31 possible influenza outbreaks since 1580, occurring approximately once every 15 years [2], with 3 occurring in the twentieth century: the outbreaks of 1918, 1957, and 1968. The 1918 pandemic influenza outbreak was the most devastating, causing between 50 and 100 million deaths worldwide [3]. In Africa, the pandemic influenza fatality count was 2.3 million deaths, which is deemed to be underreported [4]. The 1957 and 1968 pandemic influenza in Africa caused about 2–3 million and 1 million excess deaths, respectively [5]. In the twenty-first century, an influenza pandemic occurred in 2009 causing 18,156 deaths globally [6].

The highly pathogenic avian influenza (H5N1) does not usually infect humans, but poses a great threat in spillover from animal to human population, often with fatal outcomes when humans are infected. Between 1990 and 2000, avian virus H5N1 actively circulated uninterrupted among migratory birds and animals in Asia, Europe and Mediterranean, thus giving the prospects for a serious influenza pandemic outbreak in humans [7].

Following these threats and the anticipation of another pandemic, the World Health Organization (WHO) requested Member States to develop preparedness pandemic plans to ensure countries are equipped to mitigate the challenges a pandemic would present. This call was timely, given the limitations of the existing global influenza surveillance and monitoring system to respond, deploy and implement activities to mitigate the impact of an outbreak [8].

In 1999, the WHO published the first guiding principles for pandemic influenza preparedness [8]. These guidelines subsequently underwent revisions in 2005 and 2009, incorporating the practical outbreak response experiences gained from outbreaks of avian H5N1 and 2009 H1N1 influenza [9, 10]. These guidelines provide a framework for organising preparedness and response actions. The WHO recommends that, as Member States develop or update their national plans, they should consider the proposed phases in the context of country-specific needs, priorities and actions.

Based on the WHO resolution issued in April 2005 [11], many countries in Africa drafted their national plans between 2005 and 2007, and subsequently used the plans to respond to the 2009 H1N1 pandemic influenza. However, there is insufficient information on how the preparedness plans were utilized during the 2009 H1N1 pandemic and the lessons that were drawn to improve responses to the next pandemic. Furthermore, since the inception of these plans into action, no study has evaluated the quality of 2009 post pandemic preparedness plans in the WHO African region. The purpose of this present study was to evaluate the completeness of the preparedness plans. We postulated that planning for a pandemic influenza is only as satisfactory as the assumptions on which they are proposed; thus studying them is necessary. Findings from this study will be used to highlight areas of the plans that need strengthening and improvement.

## Methods

We searched the electronic databases of the WHO and United Nations (UN) plus grey literature for the availability of the national pandemic influenza preparedness plans from the WHO African region that were published between 2005 and 2017. In instances where the plans were not available online, we contacted the Ministries of Health in the respective countries for their plans. We considered countries that had plans for avian or human influenza, or both. We excluded plans that were not in public domain. Pandemic influenza plans are a blueprint for managing the emergency outbreak and, as such, should be shared with citizens and stakeholders to inform them about their roles and responsibilities in responding to a possible threat [12].

We translated plans written in French into English using google translation software. Where two national plans for a country were available, we read, assessed and treated both the draft and updated version of the plan as a unit. Four assessors (TK, CJI, CDI and AJ) independently read and scored the plans; disagreements or discrepancies that arose during assessment were resolved by a fifth and sixth reviewer (EZS and CSW).

A standard checklist with 61 binary indicators (“yes” or “no”) was used to assess the quality of the preparedness pandemic plans. The checklist, shown in Table 1, is grouped across seven thematic areas: preparation (16 indicators); coordination and partnership (5 indicators); risk communication (8 indicators); surveillance and monitoring (7 indicators); prevention and containment (10 indicators); case investigation and treatment (10 indicators) and ethical consideration (5 indicators).

**Table 1:** Standardized checklist and scores for 61 indicators grouped across seven categories

INDICATORS		RATIONALE	SCORES	
		Additional assessment guide	Number of countries	
			Yes	No
PREPARATION				
1	Does the country have a national pandemic influenza plan?	Is it publicly available?	35	0
2	Does the national influenza plan target human or avian influenza subtypes?	Human influenza subtype e.g. H1N1 and animal subtype e.g. H5N1	32	3
3	Does the national pandemic influenza plan meet the international (WHO/IHR etc) guidance on preparedness?	Is the plan based on the six phases of planning and response?	22	13
4	Are the responsibilities and actions in the plan defined phase by phase?	This is required for capacity setting, planning and command based on WHO recommendations.	21	14
5	Are there local plans at district and regional level?	See if are there any arrangements in place	9	26
6	Are business continuity plans available across the non-health sectors at national and regional levels? Or are these mentioned in the plans?	Check this among institutions (UN organization and churches etc). Do these plan mention how they will cope with an influenza pandemic and continue to provide other essential health services.	7	28
7	Are the plans flexible?	Does the plan have a severity index or are they able to adjust whether to mild or severe nature of the pandemic?	13	22
8	Do the response and inter-wave planning phases have their own courses of action and budgets which would be implemented?	These tasks should have financial and human resource with a budget provision for a year. Also see question 4	24	11
9	Is the plan sustainable for a longer term?	Influenza funding and development of command structures should not heavily rely on external funding.	0	35
10	Does the plan have a national committee(s) or advisory body in place to oversee preparedness?	Check who drafted the plan and if they were part of the committee.	32	3
11	Does the plan have any assumptions on which the plan is based?	Does the plan mention the expected range of cases and percentage of staff off sick? Check for detailed assumptions and planning principles such as case scenarios that will trigger responses and guide effective implementation of the plan.	14	21
12	Are there a national command and control structure?	This is where data or information is aggregated for the country. The national command centre exercise authority and can designate responsibilities at the local or regional levels.	25	10
13	Are there health services command and control structure?	Check for hospital and clinic plans	8	27
14	Does the pandemic plan regularly and systematically get tested at all levels and across all sectors i.e. national level health sector exercises or drills?	Check if they carry out simulations and tabletop exercises- this is important because it can feedback in the planning as lessons learnt.	8	27
15	Have the legal implications of travel restrictions and other interoperability issues been determined?	Are there any discussions or agreements on a list of issues such as cross-border management and quarantine?	15	20
16	Do interventions proposed in the plan have exit strategies?	What are the exit options? When should the pandemic be outbreak declared over?	4	31
COORDINATION AND PARTNERSHIPS				
17	Are there any regional or local arrangements in place on how to respond?	Do plans engage local people, families and medical personnel to ensure local services are running smoothly during the pandemic period?	24	11
18	Are there a regional/local planning and coordination structure?	Check for leadership roles and designation of responsibilities among the coordinating structures.	24	11
19	Is the health sector well connected to other sectors such as businesses and civil society?	Private and public partnership necessary to continue providing essential services such as water, energy and safe transport.	12	23
20	Are there joint cooperation and partnership with the neighboring countries on mutually relevant influenza policy areas?	A pandemic outbreak has no borders- check how transborder problems related to pandemic influenza will be resolved or if it is a priority in the plan.	10	25
21	Does the partnership or coordination involve financial and technical support?	This is important for planning continuity purposes and future responses.	16	19
RISK COMMUNICATION				
22	Are they a national communication strategy or is it publicly available?	Has the national communication strategy been published?	22	13
23	Does the national communication strategy sufficiently stress the likely nature or duration of the pandemic, its spread, its peak and decline, nor does it sufficiently inform the public on these issues?	Is the national communication strategy committed to public awareness including communicating the nature, spread, peak and decline of influenza (seasonal and pandemic?	11	24
24	Are there any Information Education and Communication (IEC) material or IEC in place or available?	Check if the plan use or intend to use multi-media communication i.e. newspapers, radio, TV, posters, magazines and social networking sites such as Facebook and Twitter	31	4
25	Are there any definitions of key target groups for specific preventive messages and protection such as health and emergency personnel within the communication plan?	Are there any public hygiene campaigns to highlight the personal public health measures during normal influenza seasons or outbreaks?	23	12
26	Are there effective programmes in place to change public attitudes and perceptions about influenza?	To avoid problems due to poor messages on preventive measures and general hygiene etc.	12	23
27	Are churches or religious groups mentioned in the plan to help communicate preparedness messages?	People are more likely to listen to a religious leaders than from health personnel.	8	27
28	Are there a nation-wide influenza guidance ‘intranet’ for health authorities respond quickly to an influenza outbreak?	Web reporting systems?	9	26
29	Is information exchanged with stakeholders?	Are conferences, meetings and forums mentioned for information exchange and sharing?	17	18
SURVEILLANCE AND MONITORING				
30	Are there surveillance systems in place for collecting and sharing of virological and epidemiological data with the WHO and other partners?	Check for Integrated Disease Surveillance Response (IDSR) and check if such data is shared?	18	17
31	Are there a national laboratory or national influenza centre (NIC) or Influenza assessment centres (IAC) for collecting epidemiological data on Influenza Like Illness (ILI) and Severe Acute Respiratory Infections (SARI)	The national laboratory capacity is important to provide timely, high quality, validated routine and diagnostic influenza data. ILI and SARI are indirect measures for influenza- and there are good indicators for pandemic preparedness.	18	17
32	If yes in 31, does the national laboratory have the capacity to perform: Virus isolation? Influenza typing? Influenza s	Check these at the national and administrative regional level.	13	22
33	Are there a PCR machine for testing/sequencing of seasonal and pandemic influenza viruses?	Relevant for monitoring viruses and for estimating additional resources that might be required to tackle pandemic influenza problem.	9	26
34	Are there a national “Early Warning” systems or Event Based Surveillance (EBS)	Are they a computerised hospital system that can readily give age-specific mortality data in real time?	6	29
35	Is the virological and epidemiological data shared with partners/WHO? Are they an influenza web reporting system?	Check if they have a FluNet and FluID reporting systems.	4	31
36	Are they a surveillance working group(s)?	A team of specialized expertise/epidemiologists to advise on the planning and response etc. See also question 10.	16	19
PREVENTION AND CONTAINMENT				
37	Are non-pharmaceutical intervention plans in place? i.e. closure of schools, ventilators, PPEs, quarantine, isolation, hygiene and sanitation.	Are prevention and cluster control plans in place (i.e. for border and stamping influenza out prior to widespread in the country.	26	9
38	Are pharmaceutical interventions in place? i.e. use of vaccines, antivirals and antibiotics for secondary infections	Check for vaccine strategy if in place?	29	6
39	Are there a procurement strategy of pharmaceutical (vaccines) and non-pharmaceutical products (PPEs)?	Check for political intervention to improve pharmaceutical logistics in acquiring vaccines and other drugs.	17	18
40	Are there contracts and agreements with pharmaceutical companies for the supply of equipment and drugs for influenza preparedness capacity?	Check if there are vaccine and antiviral drug contracts and agreements with the pharmaceutical companies.	2	33
41	Are there a pharmaceutical (vaccine) strategy	If a pandemic vaccine is planned to be used when will the vaccines arrive in health centres? Is it within six months of the start of the pandemic?	12	23
42	Are there accelerated regulatory approvals of influenza vaccines for quick deployment? Or are there a national regulatory capacity in place so that vaccines, diagnostic tests and antiviral medicines for influenza can be deployed quickly?	Some countries deploying influenza vaccines are required to meet the preconditions for supply of vaccines through the WHO Deployment Initiative.	3	32
43	Are there any additional (surge) capacity to improve responses through training and increasing human resource capacity?	Are there a standardised national educational materials for all health care workers?	21	14
44	Are there effective hospital control policies?	Do hospitals or health centres have their own plans?	5	30
45	Are there plans for recruiting volunteers from local communities?	This is necessary in case of staff absenteeism during the pandemic period.	2	33
46	Are there a reserve list of health professionals?	Necessary in case of staff absenteeism during the pandemic period.	4	31
CASE INVESTIGATION AND TREATMENT				
47	Are there any scientifically-based estimates of the numbers of people likely to be affected by pandemic influenza and needing medical and social care?	These estimates contributes to the planning of resources and for efficient and equitable deployment of vital supplies for pandemic influenza.	8	27
48	Are there a list of critical information that is needed early in a pandemic (e.g. attack rates by age and locality, strain type, likely antiviral sensitivity, response to antivirals and public health measures, etc)?	What is the proportion of the population that may need treatment i.e. target groups for prophylaxis?	9	26
49	Are there criteria for the types and amounts of antivirals to be used?	Does the plan have priorities on the types of antivirals or drug combinations?	18	17
50	Are there a local distribution channel to deliver these antivirals and vaccines?	Hotlines e.g. telephone lines for requests and local influenza centres to deliver.	13	22
51	Are there any consideration of mechanisms to monitor the usefulness of vaccines, effectiveness, side-effects and resistance of antivirals through real time surveillance?	Necessary for efficient and timely decision-making	8	27
52	Are border screenings in place and will the cases be followed-up?	Contact tracing e.g. interviewing patient cases and carrying out surveys for possible sources?	15	20
53	Are isolation or quarantine rooms provided at the port of entry?	Rooms to hold suspected cases.	16	19
54	Are there a national annual seasonal influenza vaccination programme in place?	Necessary if countries will be able to vaccinate timely during the pandemic period.	0	35
55	If yes it is achieving > 75% uptake in over 65 s and increasing uptake in occupational and clinical risk groups?	Vaccinating the elderly and at risk adults, for example, is unlikely to establish indirect protective effects because these groups represent a small percentage of the population among whom the virus spreads.	0	35
56	Are there vaccine uptake figures or are these published annually?	If the vaccine uptakes are low, are there plans in educating the public on attitudes and perceptions?	0	35
ETHICAL CONSIDERATIONS				
57	Is there an ethical framework in place?	Necessary to avoid ethical problems that might arise	1	34
58	Are there any ethical consideration for appropriate use of quarantine procedures, treatment of patients with vaccines and antiviral drugs?	Are there priority setting and equitable access to therapeutic and prophylactic measures? What are the core governmental responsibilities on this?	4	31
59	During implementation of the plan, are there consideration to balance public health and human rights?	During a pandemic influenza emergency, policymakers experience tension and disputes, and that they struggle to balance public health decisions between what is best for the individual and society as a whole.	6	29
60	Are there evidence base for public health measures on which decisions will be based or are based?	Check in the plans if policymakers use science	6	29
61	Are there transparency, public engagement and social mobilization in the plan?	Is there a list that shows the beneficiaries for the interventions or how the beneficiaries were selected as eligible candidates for the interventions or limited resources?	7	28

The indicators used to assess the African plans were developed partly from the 20 key indicators on various goals of preparedness recommended by the European Centre for Disease Prevention and Control (ECDC) and WHO Regional Office for Europe [13]. A group of 25 European countries plus Iceland and Norway through a consultative process provided feedback on the content validity of the 20 indicators [13]. Additional indicators specific to the purpose of our study and setting was pulled together by incorporating other recommendations from the WHO guidance on pandemic plan development [13, 14]. The final instrument was validated by pandemic policy planners in 7 select countries with a validity index score of not less than 0.75.

Each plan assessed would score a maximum of 61 points for completeness across the 7 thematic areas of preparedness. We generated descriptive data, such as averages and percent of total, to gauge quality of pandemic preparedness plans. An overall plan score was calculated by assigning 1 or 0 points to each indicator. An indicator score of one is assigned to the plan if denoted by “yes” and zero for “no”. The indicator was scored 1 if an item was mentioned in detail or partly described in the plan, while a score of 0 was given if the item assessed was missing or absent in the plan. All the scores were verified before entry in excel by two reviewers (EZS and CDI) prior to analysis.

## Results

Of the 47 countries in the WHO African region, 35 national pandemic plans were retrieved for assessment in this study (Table 2). We could not find plans for 12 countries- either they were not publicly available or we could not access them from the Ministry of Health in these countries upon request.

**Table 2:** Country pandemic plans assessed, year of development and last updated

	Country	Year		Country	Year
1	Algeria	2009	19	Madagascar	2006
2	Benin	2006/2009	20	Malawi	2006
3	Botswana	2005	21	Mali	2006
4	Burkina Faso	2005	22	Mauritania	2006
5	Cameroon	2006	23	Mauritius	2006
6	Cabo Verde	2006	24	Mozambique	2006
7	Central African Republic (the)	2006	25	Namibia	2005
8	Chad	2006	26	Niger (the)	2006
9	Comoros (the)	2006	27	Nigeria	2007
10	Côte d’Ivoire	2009	28	Rwanda	2006
11	Democratic Republic of the Congo (the)	2006	29	Senegal	2005/2009
12	Gabon	2007	30	Seychelles	2007
13	Gambia (the)	2006/2009	31	Sierra Leone	2005/2009
14	Ghana	2005/2009	32	South Africa	2006/2017
15	Guinea	2006/2009	33	Swaziland	2006
16	Kenya	2005	34	Uganda	2006
17	Lesotho	2006	35	United Republic of Tanzania (the)	2007
18	Liberia	2009			

**Fig. 1:**
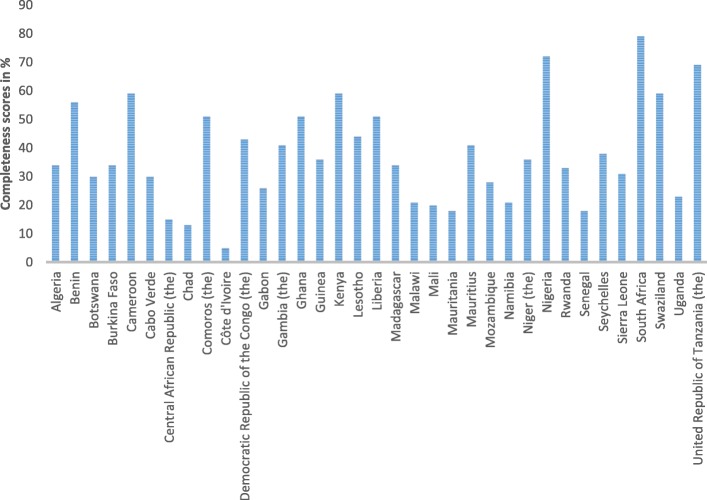
Composite scores of preparedness plans by country

Of the plans reviewed, 60% were initially developed between 2006 (Table 2) in response to specific threats posed by the continuing spread of the avian influenza (H5N1) virus. Figure 1 shows composite scores of preparedness plans by country. The composite score for the completeness of the pandemic plans was 36% across the 35 countries. Country-specific scores on each of the thematic indicators for pandemic plan completeness varied, ranging from 5% in Côte d’Ivoire to 79% in South Africa (Fig. 1). Overall, our assessment shows that pandemic plans across the WHO African region remain inadequate, with no details on ethical considerations, case investigation and treatment. Nigeria was the only country that scored 60% across all the thematic areas of preparedness.

**Fig. 2:**
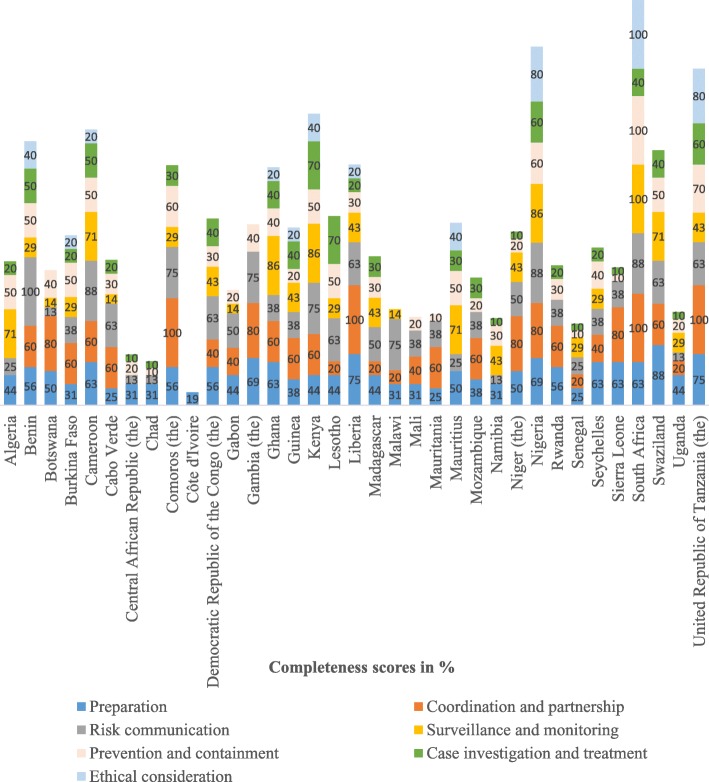
Completeness of the preparedness plans of countries by category

Figure 2 shows completeness of the preparedness plans of countries by thematic area. On average, preparation and risk communication scored 48%, respectively, while coordination and partnership scored highest with an aggregate score of 49%. Surveillance and monitoring scored 34%, while prevention and containment scored 35%. Case investigation and treatment scored 25% and ethical consideration scored the lowest of 14% across 35 countries.

Table 1 shows the scores of the assessment indicators for all thematic areas. Of the countries that had a plan available online, 33 countries planned against both human and avian influenza subtypes. Three plans- those from Algeria, Chad and Cote d’Ivoire- specifically focused on the planning for and response to avian influenza subtypes. 22 of 35 plans followed the WHO guidance on six phases of planning and response. 14 countries cited hypothetical scenarios on which the plan is based, for example, when doses of vaccines and antivirals need to be acquired to treat patients. There were 9 plans with planning initiatives at the district and regional levels, and 7 plans mentioned that they had business continuity plans across the non-health sector. We found 13 plans to be flexible with regards to the ability to quickly adjust to the severity of the pandemic. 24 countries had a budget provision for each course of action, however, all the plans were heavily dependent on external funding with no sustainable budget for their preparedness. Maximum funding for some countries, such as the Democratic Republic of Congo, was only 3 years. All but 3 countries- Algeria, Cabo Verde and Central African Republic- mentioned having a national committee or advisory body to oversee preparedness. Eight plans tested their planning for and responses through exercises and drills at the national level. There were 25 plans that had a national command and control structure, where influenza data or epidemiological information is aggregated and shared with regional and district levels. Hospital plans were available in 8 plans and only 4 countries had planned for exit strategies after the pandemic.

Coordination and partnership indicators showed that 24 plans engaged local people, families and medical personnel to ensure local services run smoothly during the pandemic. Another 24 plans had a functional local or regional coordination structure. 12 countries had a private and public partnership to offer essential services such as the delivery of health, safety and energy. Ten national plans had a joint cooperation and partnership with a neighbouring country on mutually relevant influenza policy. 16 countries held partnership and coordination that involved financial and technical support.

The risk communication indicator showed that 22 plans had a communication strategy and 11 plans mentioned the role of public awareness, including sharing information on the nature, transmission patterns, peak and decline of the influenza. 31 plans had Information, Education and Communication (IEC) materials published in multi-media such as newspapers, radio, television and social networking sites on the internet. 23 plans defined key target groups for specific preventative messages, such as public hygiene campaigns to highlight the personal public health measures during normal influenza seasons or outbreaks. 12 plans planned to avoid problems arising due to poor communication around preventative measures and general hygiene. Only 8 plans mentioned churches or religious groups to assist with communicating messages on preparedness. 9 countries had web reporting systems, such as intranet or FluNet, to speed up responses to an influenza outbreak. Information exchange among stakeholders through conferences, meetings and forums were mentioned by 17 plans.

Surveillance and monitoring are considered an important part of planning, yet 17 plans failed to mention the surveillance techniques of collecting and sharing influenza virological and epidemiological data. This is despite the presence of the integrated disease surveillance response (IDSR) system in many African countries. In these countries, there was no national influenza centre (NIC) or influenza assessment centres (IAC) for collecting epidemiological data on influenza-like illnesses and severe acute respiratory infections. Amongst those that had a laboratory, 13 countries had the capacity to perform virus isolation, typing and subtyping. 9 countries had a polymerase chain reaction (PCR) machine to test and monitor influenza circulation. Only six plans had a computerised hospital system as an early warning system that can readily give real-time data on influenza outbreaks. Epidemiological and virological data was shared with the WHO and other partners by 4 countries- Algeria, Ghana, Kenya and South Africa. There were 16 plans that mentioned having a surveillance working group to give advice on surveillance and monitoring.

As part of prevention and containment of influenza, 26 countries planned for non-pharmaceutical interventions, such as closure of schools, use of ventilators, use of personal protective equipment, quarantine, isolation, hygiene and sanitation. In terms of pharmaceutical interventions, 29 plans mentioned strategies that would use vaccines, antivirals and antibiotics for treatment of secondary infections. With regards to detailed assessment of the pharmaceutical strategy, we found that 12 plans had a vaccine strategy, while 17 plans had a procurement strategy for either pharmaceutical or non-pharmaceuticals products. Only 2 plans, those from the United Republic of Tanzania and South Africa, had advanced contracts and agreements with pharmaceutical companies in place for the supply of equipment and drugs for influenza treatment. 3 plans, those from the United Republic of Tanzania, Swaziland and South Africa, had in place accelerated regulatory approval of influenza products for quick deployment. Additional surge capacity to improve responses through training and human resources was available in 21 of the plans. The hospital plans were available in 5 plans and 2 plans (Algeria and South Africa) mentioned the need for recruiting volunteers from the local community. In terms of human resource, 4 plans suggested recruitment of staff from a reserve list of health professionals.

In the category of case investigation and treatment, 8 plans had science based influenza planning assumptions for efficient and equitable deployment of vital supplies against influenza. As part of planning, 9 plans included critical information such as attack rates by age and locality, strain type, antiviral sensitivity or who to target for prophylaxis. 18 plans mentioned the criteria and types of antivirals to use in an event of an outbreak. The most commonly mentioned antivirals were zanamivir and oseltamivir. About 13 plans mentioned that they will deliver these antivirals through local distribution channels, including the use of telephone line and local influenza centres. Mechanisms to monitor the effectiveness, side effects and resistance of vaccines or antivirals were considered in 8 plans through real time surveillance. Plans to screen cases at the borders and follow up cases were indicated in 15 plans, while isolation or the provision of rooms at the border entry were only mentioned in 16 plans. No plan reported the intention to vaccinate seasonally (i.e. achieving > 75 uptake in the elderly population), nor published any vaccination figures despite indicating that they will vaccinate its population.

Ethical consideration was inadequately reported in most plans, with only 1 plan (South Africa) having completely reported to have an ethical framework in place. 4 plans considered an ethically appropriate use of quarantine procedures, fair allocation of treatment and limited resources such as vaccines. 6 plans considered how to balance between public health and human rights interests if they came into conflict. 7 of the plans indicated the need for transparency in decision making, for example, how eligible beneficiaries would be selected to receive scarce interventions.

## Discussion

Preparing for a response towards a pandemic extends beyond the development of the plan to include an implementation plan that lays out how the goals of the plan match available resources, tasks and responsibilities, to meet the needs of the population affected by the pandemic outcomes. Preparedness plans are crucial to build frameworks for emergency response, thereby providing countries with the opportunity to plan, strategise and mobilise human and capital resources before a pandemic occurs. Adequate and thorough plans ensure that countries can respond immediately when a pandemic is declared.

While our study showed that the majority of the African countries have a plan (74%), the majority of these plans are inadequate, with many tasks necessary to address pandemic threats of the twenty-first century remains unmet. This finding corresponds to studies that evaluated preparedness plans and responses to the 2009 H1N1 pandemic in Ghana and Malawi, where such plans were found to be weak and unable to elicit the most desired responses during the pandemic [15, 16]. The findings of this study also concur with an evaluation done by Ortu et al. (2008), who reported that the plans lacked operational clarity and focus of the planning objectives [17].

Our findings indicate that the majority of plans have not been updated over time, despite the lessons offered by the 2009 H1N1 pandemic. Our findings also show that only 7 of the assessed countries in Africa updated or revised their plans periodically to incorporate the changing circumstances and lessons gathered from the 2009 pandemic. For instance, South Africa is one of the countries with consistent updates to its plan, with a recently developed five-year national influenza policy and strategic plan outlining a comprehensive approach to influenza prevention and control [18]. A plan needs to be a living document, periodically adapted as new information on the influenza becomes available and thus ready to provide a guide to the protocols, procedures, and division of responsibilities in emergency response [12].

Results of our study also suggest that many countries did not consider the proposed phases of preparedness to respond more efficiently to the influenza pandemic. This is despite the fact that the WHO has provided an up-to-date evidence-based guidance to support countries to develop and revise pandemic preparedness plans. Recently, the WHO published an updated pandemic influenza preparedness checklist to help Members States build capacity for pandemic response [14]. However, our review highlights how many countries in the WHO African region are yet to incorporate these guidelines despite the need to improve existing plans.

Our study also shows that many countries do not have business continuity plans across the non-health sector at the subnational level. An influenza pandemic is an unpredictable event that can create a major management crisis of unprecedented scale and cost. High absence of workers from duty could drastically interrupt the functioning of critical infrastructure, such as services essential to health, technology and communication networks, economic wellbeing, safety and security. Due to the disruptive nature of the pandemic to social services and the economy, development of business continuity plans embedded within the national plan is critical for an effective country response that minimizes the financial consequences on all businesses of all sizes and types [19].

In our study, we observed that only a few national plans engaged with specific sectors, such as education, hospitals, industry and local community. It is useful for plans to make meaningful arrangements at the local level, because this is where the burden of the disease occurs and is largely felt. In addition, in the aftermath of the pandemic, the local level is where the plans can continue to be implemented. Interestingly, apart from local coordination, we found that few countries had joint cooperation and partnership from non-health sector in preparedness, thus making interoperability and integration of planning efforts and services impossible. The purpose of planning and involving cooperation and partnership at all levels is to support and promptly restore key routines and functions prone to disruptions in our societies. Even a well-designed and motivated plan without partnerships will fall short in managing the crisis, and will struggle to guide recovery effectively if it does not extend responsibilities and command across local government, stakeholders and international partners.

Although surveillance is considered one of the most crucial planning activities, in this study we found that half of the plans did not incorporate the techniques of collecting virological and epidemiological data for the early detection of the virus causing an epidemic. The majority of the surveillance plans in place were weak. The role of surveillance techniques and systems is to send early signals of an imminent influenza outbreak in the human and animal population, and yield knowledge for treatment, prevention and control of influenza [20]. For many plans, it was impossible to fulfill these tasks in the absence of laboratories and equipment, such as PCR machines to perform virus typing and subtyping. According to the IHRs, all countries are mandated to monitor and rapidly report disease outbreaks that pose a threat to other countries [11]. Apart from alerting respective countries about the nature of the influenza virus in circulation, understanding disease virology can be useful for vaccine production. However, without the necessary tools to conduct surveillance, public health interventions to reduce influenza pandemic are jeopardized.

An interesting finding from this study was that 26 countries proposed to use non-pharmaceutical interventions (case isolation, restricting children’s visits to hospitals, workplace closure etc.), while nearly all indicated the use of pharmaceutical interventions i.e. vaccines, antivirals and antibiotics for treatment of secondary infections. Although vaccines are a primary strategy for preventing and mitigating influenza outbreaks, many plans do not specify whether vaccines will be acquired on time. Since influenza viruses change overtime due to the antigenic shifts and drifts, it is difficult to produce an appropriate and effective influenza vaccine for unknown subtypes [20].

As such, during the first few months of a pandemic influenza, vaccination will not be a primary intervention strategy. The time during which there are no vaccines, combined approaches of non-pharmaceutical interventions can minimize morbidity and mortality due to influenza pandemic. There is no point in making arrangements to use vaccines (including other treatments products and materials) when these products will not be available or are unlikely to be supplied within a useful time frame to mitigate the disease. If specific arrangements are proposed, then plans should take into account both the limitations and the capabilities of the responses.

Most importantly, although often forgotten in the majority of the preparedness plans is the need for ethical considerations. Our study indicates that, with the exception of one plan (South Africa), no other plans reported having an ethical framework in place. There is an expectation that during a pandemic influenza outbreak, ethical issues will arise due to conflicting interests between civil liberties (i.e. violation of human rights) and population health (i.e. greatest good for the greatest number) [20]. In the absence of an ethical plan, it is difficult to respond appropriately to ethical dilemmas and this can constitute a threat to preparedness and response. We propose that all countries develop an ethical framework that can be used to address ethical problems such as these of rationing limited vaccines or failure by health care-workers to work on the bedside during the pandemic.

Our study has several limitations. Our analysis was based on pandemic plans that are freely available online and thus it is possible that some of these plans would have been updated and the revised versions of the plans not yet published. Our study may therefore be a misrepresentation of the preparedness. We were only able to assess written materials in the protocols, yet crisis preparedness extends beyond these documents to include the ability to perform within the means using the necessary and available tools and infrastructure. Thus, we are not suggesting that countries that scored high in the completeness scores for preparedness will do the same in real crisis situations. However, for country preparedness to be truly effective at preventing and responding to influenza, plans must be created and drills and exercises conducted to ensure they prevent and address influenza pandemic. Another limitation involved the process of scoring the plans without a weighting scale, which may have introduced bias especially among those indicators that fell between 1 and 0. A further methodological limitation involved scoring the same plan twice i.e. the initial draft and updated version. As such, countries with more than one national plan may have been more likely to achieve a higher score, thus skewing the scores for those plans. Finally, we used google translation software to translate French plans into English and thus some words may have been lost in translation. Most importantly, we excluded one French written plan (Togo) from the analysis because the format of the plan made it unable to be translated.

## Conclusion

Based on our assessment of the plans, we found preparedness plans to be weak therefore, these plans must address the gaps identified in this study. We recommend improving the overall goals in preparedness and these are achievable through drills, simulations and tabletop exercises.

## Abbreviations

EBS: Event based surveillance
ECDC: European centre for disease prevention and control
H: Hemagglutinin
HPAI: Highly pathogenic avian influenza
IAC: Influenza assessment centres
IDSR: Integrated disease surveillance response
IEC: Information, education and communication
IHR: International health regulations
ILI: Influenza like illness
N: Neuraminidase
NIC: National influenza centre
PCR: Polymerase chain reaction
SARI: Severe acute respiratory infections
WHO: World Health Organization
